# Cornelia de lange syndrome

**DOI:** 10.4103/0971-6866.42324

**Published:** 2008

**Authors:** Naeimeh Tayebi

**Affiliations:** Medical Doctor-Genetic Counselor, Genetic Research Center-Shahid Fiazbakhsh Rehabilitation Comprehensive Center-Yazd Welfare organization

**Keywords:** Cornelia de lange syndrome, distinctive facial features, long philtrum, malformation of upper limbs, Synophrys

## Abstract

**BACKGROUND:**

Cornelia de Lange syndrome (CDLS) is a rare multiple congenital anomaly syndrome characterized by a distinctive facial appearance, developmental delay, growth retardation, low birth weight, skeletal formation anomaly, and hirsutism.

**CASE:**

Here for the first time a case of CDLS from Iran, a 15-week-old male infant who was refereed as a case of multiple congenital anomalies. Clinical investigation showed that the child was a case of CDLS.

**CONCLUSION:**

This is the first case report with CDLS in Iran.

## Introduction

Cornelia De Lange syndrome (CDLS) was first described by Cornelia de Lange, a Dutch pediatrician in 1933.[1] This syndrome is also called as Brachmann de Lange syndrome (BDLS) since he reported a patient with similar symptoms at autopsy in 1916.[2]

Incidence of this syndrome is variable, ranging from 1:10,000 to 1:100,000 live births in different population groups.[3] There is no racial predilection. It is slightly more common in females as compared to males (F/M: 1.3/1). Most children with this syndrome could not live more than 2 years and the main cause of death was pneumonia along with cardiac, respiratory, and gastrointestinal abnormalities.[4]

CDLS is a multisystem developmental disorder characterized by growth and developmental retardation, low birth weight, hirsutism, anomalies in the structure of the upper limbs, gastroesophageal dysfunction, ophthalmologic and genitourinary anomalies, congenital diaphragmatic hernia, cardiac septal defect, distinctive facial features, learning difficulties, and mental retardation.[5] As a rule, they fail to thrive.

The facial characteristics are the most diagnostic, with microcephaly, the neat, well-defined, and arched eyebrows growing across the base of the nose (synophrys or confluent eyebrows), long curly eyelashes, short neck with low anterior and posterior hairlines, long philtrum, generalized hirsutism, thin lips, micrognatia, a small nose with low bridge, low set ears, and crescent-shaped mouth.[6]

The genetic and molecular bases of this syndrome are not clear. However, it is thought to be the result of a dominant mutation.[7] A large part of the cases diagnosed as CDLS seem to be sporadic and 10% of the cases present chromosomal alterations, such as a small duplication of the long arm of chromosome 3[8] or unbalanced chromosomal rearrangement.[9]

In this article, we have reported for the first time a case of CDLS from Iran, which has been investigated at the Genetic department at Yazd Welfare organization.

## Case Report

A 15-week-old male infant was refereed to Genetic Research Center of welfare organization, Yazd, Iran in Oct 2007 on account of multiple congenital anomalies.

He was a child of nonconsanguineous marriage, born of a full-term normal delivery with a birth weight of 1800 g, height of 47 cm and head circumference of 30 cm. The present weight, height and, head circumference were 2700 g, 49, and 39 cm, respectively. Also, he was the second child. The first child was a 6-year-old boy who was normal. Both parents were normal and there was no history of deformity in their pedigree.

He was confined to bed during 18 days after birth because he had feeding problems and gavage feeding was done for him.

**Figure 1:**
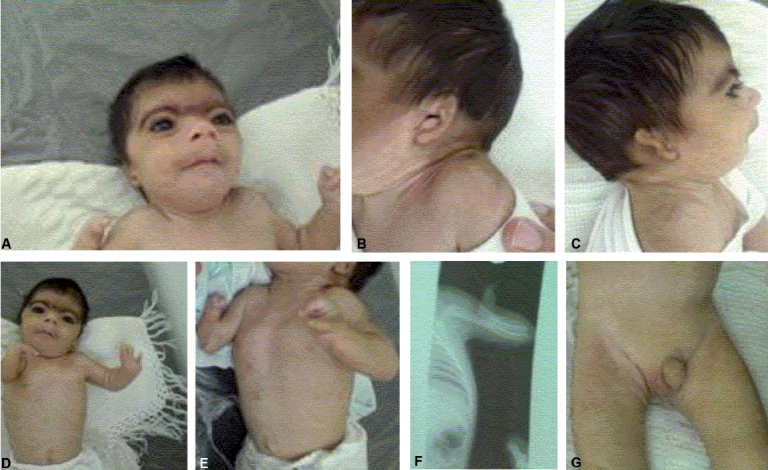
A 15-week-old male infant with Cornelia De Lange syndrome

At presentation, the patient had confluent eyebrows that appeared arched and well-defined and bushy, long curly eyelashes, low front and back hairlines, small nose with low bridge, turned-up nose, down-turned angles of the mouth and thin lips, long philtrum, highly arched palate, small lower jaw and protruding upper jaw, microcephaly [Figure 1A], to wide Anterior and posterior fontanelles, excessive body hair, Cutis marmorata, facial hypertrichosis, and malformed pinnas [Figure 1B, C]. The patient had a low-pitched cry, umbilical hernia was seen when he cried, short neck, fixed flexion of both elbows [Figure 1D,E].

An X-ray of right hand showed ulnar agenesis and monodactyl [Figure 1F], but the X-ray of left hand showed that both forearm bones were normal, but there were three fingers. His feet were not malformed.

Long coarse black hairs were present on the back associated with generalized hypertrichosis since birth.

The baseline blood, urine investigations and thyroid function tests were within normal limits. The cardiovascular, respiratory, brain computed tomography (CT) scan and ophthalmologic examination were described as normal. Ultrasonography of the abdomen did not reveal any organomegaly.

Examination of the genitalia showed bilateral undescended testis with hypospadiasis [Figure 1G].

## Cytogenetic analysis

Peripheral blood from the patient was subjected to short-term culture in RPMI 1640 medium for 72 h. After metaphase arrests through exposure to Colcemide, cells were harvested, treated with hypotonic solution, and then fixed with methanol and acetic acid according to standard procedures.[10] The harvested cells were dropped on clean slides and G-banding was done, and then stained with Wright's stain, for chromosome banding.[10] The clonality criteria and the karyotypic descriptions were according to the ISCN recommendations.[11] Analysis of 25 metaphase cells showed 46, XY in all cells.

## Discussion

CDLS is relatively uncommon multiple congenital anomaly with unknown cause and recurrent risk. This syndrome may be the result of an inheritance metabolic error.[12] No environmental cause has been discovered. Although an autosomal dominant, autosomal recessive, and chromosomal anomaly have been suggested, most cases are sporadic.[13]

Diagnosis of CDLS is dependent on the recognition of distinctive facial features,[14] in addition to the physical features as pre- and postnatal growth retardation, microcephaly, feeding problems, major malformations including limb defects, and characteristic facial features.[15]

Due to the characteristic facial features, the physiological findings, and the presence of a normal karyotype, the patient was diagnosed as CDLS.[16]

Physiological findings to support the diagnosis were microcephaly, excessive body hair, short neck, flexion of both elbows, absence of right ulna, malformed pinnas, and bilateral undescended testis with hypospadiasis.

Chromosomal analysis was performed to find out if there were chromosomal imbalances or gross rearrangement of the drosophilia nipped B gene (NIPBL) or SMC1L1 gene regions. The karyotype was normal. Analyses for mutations in these genes are not currently available in Iran and there were no funds for testing abroad. Nonetheless, based on the clinical features, the present patient was believed to be the first CDLS case reported in the Iran.

Ellaithi *et al.* reported a case of BDLS from Sudan for the first time. The patient was a 7-month-old female infant, who was refereed as a case of malnutrition. Clinical investigation showed that the child was a classical case of BDLS.[17]

Kim *et al.* evaluated ophthalmologic problems, for the first time, in a case of CDLS. They presented a case of CDLS in an 18-year-old female with a superficial keratitis with entropion, ptosis, high myopia, lacrimal cutaneous fistula, and facial appearance.[18] Also, Badoe, Grau Carbó *et al.*, Muhammed *et al.* described cases with clinical features of CDLS.[19–21]

## Conclusion

CDLS is a rare but well-characterized syndrome. The key diagnostic features are the distinctive facial features, limb anomalies, and growth retardation. It is hoped that with the report of this case at the Genetic Research Center, Welfare organization, Yazd, Iran every attempt will be made to characterize “multiple congenital anomalies” that present to this center.
